# Central Role of Glutamate Metabolism in the Maintenance of Nitrogen Homeostasis in Normal and Hyperammonemic Brain

**DOI:** 10.3390/biom6020016

**Published:** 2016-03-26

**Authors:** Arthur J. L. Cooper, Thomas M. Jeitner

**Affiliations:** Department of Biochemistry and Molecular Biology, New York Medical College, Valhalla, NY 10595, USA; Thomas_Jeitner@nymc.edu

**Keywords:** Amino acids, ammonia, aspartate, aspartate aminotransferase, glutamate, glutaminase, glutamate dehydrogenase, glutamine, glutamine synthetase, α-ketoglutarate

## Abstract

Glutamate is present in the brain at an average concentration—typically 10–12 mM—far in excess of those of other amino acids. In glutamate-containing vesicles in the brain, the concentration of glutamate may even exceed 100 mM. Yet because glutamate is a major excitatory neurotransmitter, the concentration of this amino acid in the cerebral extracellular fluid must be kept low—typically µM. The remarkable gradient of glutamate in the different cerebral compartments: vesicles > cytosol/mitochondria > extracellular fluid attests to the extraordinary effectiveness of glutamate transporters and the strict control of enzymes of glutamate catabolism and synthesis in well-defined cellular and subcellular compartments in the brain. A major route for glutamate and ammonia removal is via the glutamine synthetase (glutamate ammonia ligase) reaction. Glutamate is also removed by conversion to the inhibitory neurotransmitter γ-aminobutyrate (GABA) via the action of glutamate decarboxylase. On the other hand, cerebral glutamate levels are maintained by the action of glutaminase and by various α-ketoglutarate-linked aminotransferases (especially aspartate aminotransferase and the mitochondrial and cytosolic forms of the branched-chain aminotransferases). Although the glutamate dehydrogenase reaction is freely reversible, owing to rapid removal of ammonia as glutamine amide, the direction of the glutamate dehydrogenase reaction in the brain *in vivo* is mainly toward glutamate catabolism rather than toward the net synthesis of glutamate, even under hyperammonemia conditions. During hyperammonemia, there is a large increase in cerebral glutamine content, but only small changes in the levels of glutamate and α-ketoglutarate. Thus, the channeling of glutamate toward glutamine during hyperammonemia results in the net synthesis of 5-carbon units. This increase in 5-carbon units is accomplished in part by the ammonia-induced stimulation of the anaplerotic enzyme pyruvate carboxylase. Here, we suggest that glutamate may constitute a buffer or bulwark against changes in cerebral amine and ammonia nitrogen. Although the glutamate transporters are briefly discussed, the major emphasis of the present review is on the enzymology contributing to the maintenance of glutamate levels under normal and hyperammonemic conditions. Emphasis will also be placed on the central role of glutamate in the glutamine-glutamate and glutamine-GABA neurotransmitter cycles between neurons and astrocytes. Finally, we provide a brief and selective discussion of neuropathology associated with altered cerebral glutamate levels.

## 1. Introduction

Glutamate is the most abundant of the common protein-coded amino acids in the brain [1]. Table 1 lists the concentration of the more abundant amino acids in cat, rat, and human brain. In addition to high concentrations of glutamate, the brain contains mM concentrations of glutamine, aspartate, and GABA (Table 1). Taurine is included in this table because, although it is not a protein-coded amino acid, its concentration in brain is high. Taurine plays important roles in the brain as an osmolyte (volume regulation), calcium homeostasis, and regulation of neurotransmission, e.g., [2,3,4]. Glutathione (GSH: a tripeptide, γ-glutamylcysteinylglycine) is also included in this table because it is synthesized from glutamate. In the brain, GSH is a major redox buffer, water soluble antioxidant (along with ascorbate), enzyme cofactor and participant in detoxification processes, especially in astrocytes, e.g., [5]. Glutamate and, to a lesser extent, aspartate are the major excitatory neurotransmitters in the brain, whereas GABA is the main inhibitory neurotransmitter. Therefore, these amino acids must be maintained at very low concentrations in the extracellular fluid compartments of the brain. For example, the concentrations of glutamate and aspartate in human cerebrospinal fluid (CSF) are ~8 and 0.2 µM, respectively [1]. The concentration of GABA in human CSF is ≤0.1 µM [6]. Interestingly, the concentration of glutamine in human CSF is remarkably high (~50 µM) and greater than that of all the other common amino acids combined [1]. In point of fact, the concentration of glutamine in human CSF is >50 times greater than that of glutamate [1]. This high concentration of glutamine is a reflection of the release of glutamine from astrocytes to the extracellular fluid as a means of maintaining nitrogen balance and as part of the glutamate-glutamine cycle hereinafter simply referred to as the glutamine cycle (Section 6).

The concentration of glutamate in synaptosomal vesicles is very high [7], perhaps as high as 100 mM or greater (ref. [8] and references cited therein), representing 15%–20% of the total glutamate pool in synaptosomes, consistent with high levels in the nerve endings [7]. The very high concentration of glutamate in the cytosol and glutamate-containing vesicles requires strict homeostatic mechanisms for the following reason. Glutamate is the major excitatory neurotransmitter, yet levels of glutamate in the extracellular fluid must be kept low (<100 µM) to avoid excitotoxicity. In fact, the concentration of glutamate in the ambient extracellular fluid of the brain is normally 0.5–5 µM [8]. This remarkable glutamate concentration gradient between the extracellular fluid and nerve cell cytosol is accomplished by powerful uptake systems for glutamate in neurons, astrocytes and synaptosomal vesicles. (For a recent review see [9]).

**Figure 1:**
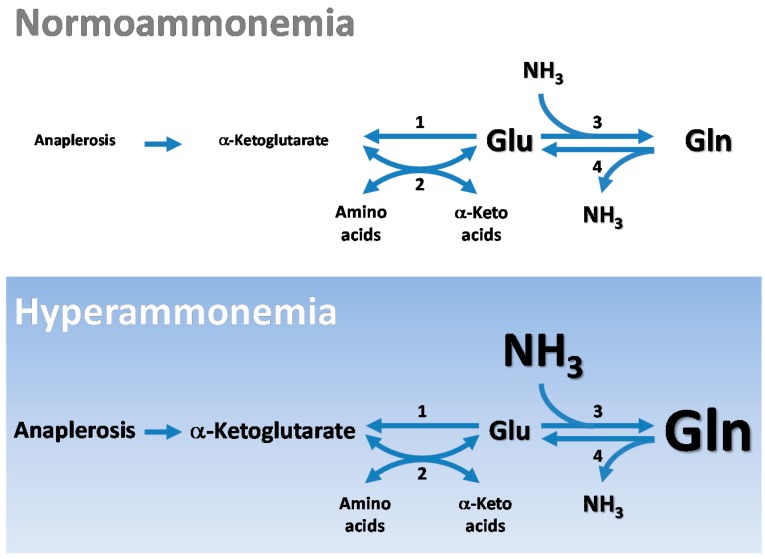
Schematic of the *major* pathways by which cerebral glutamate levels are maintained during normoammonemia (**top panel**) and hyperammonemia (**bottom panel**). Relative changes in pool size of cerebral metabolites (α-ketoglutarate, ammonia, glutamate, and glutamine) between nomoammonemic and hyperammonemic brain are indicated by differences in font size. Enzymes: **1**, glutamate dehydrogenase (GDH); **2**, α-ketoglutarate/glutamate-linked aminotransferases (notably aspartate aminotransferase (AspAT) and branched-chain aminotransferases); **3**, glutamine synthetase; **4**, glutaminase. Note that despite the ammonia-stimulated increase in glutamine (Gln), glutamate (Glu) levels are only modestly depleted. This is in large part due to hyperammonemia-induced increase in the activity of anaplerotic enzymes including pyruvate carboxylase. Note also that for simplicity not all reactants are shown in the enzyme-catalyzed reactions.

Exclusion of most blood-borne glutamate at the blood-brain barrier (BBB) and a net removal of glutamine from the brain (see below) indicate that the cerebral pools of glutamate are largely produced within the brain. Thus, the tricarboxylic acid (TCA) cycle must be an important source of 5-carbon units for the synthesis of the glutamate backbone in the brain. The nitrogen of the glutamate pool in the brain is mostly supplied by aminotransferase reactions. Here, we review the central importance of aminotransferases in maintaining nitrogen homeostasis in the brain, with special emphasis on glutamate/α-ketoglutarate-linked aminotransferases. We highlight the important role of glutamate as a precursor for other important metabolites including GSH. We also emphasize the special role of glutamate in the glutamine- and glutamine-GABA cycles of the brain. The glutamate utilized in these pathways is maintained at a remarkably constant level. For example, during hyperammonemia there is a marked increase in the rate of conversion of cerebral glutamate to glutamine, yet although there is some depletion in glutamate the decrease is not stoichiometric with the concomitant large increase in glutamine concentration. There is also no change in the concentration of cerebral α-ketoglutarate or a modest increase. Thus, there is a considerable net increase in the concentration of 5-carbon units (*i.e.*, glutamate plus glutamine plus α-ketoglutarate) resulting from an ammonia-induced stimulation of anaplerosis. An especially prominent anaplerotic enzyme stimulated by hyperammonemia is pyruvate decarboxylase. The main pathways of glutamate metabolism in normoammonemia and hyperammonemia are depicted diagrammatically in Figure 1.

Note that throughout the text we use the term ammonia to refer to the sum of ammonium (NH_4_^+^) ions and ammonia free base (NH_3_). Since the p*K*_a_ of ammonia is ~9.2 at physiological pH values (7.2–7.4) only ~1% of ammonia will be in the form of NH_3_.

## 2. Glutamate Formation in the Brain—Important Roles of Glutaminase and α-Ketoglutarate-Linked Aminotransferases

An important route for the formation of glutamate carbon and nitrogen in the brain is via the glutaminase reaction. In this section we describe the reason why the glutaminase reaction and not the GDH reaction is an important source of brain glutamate. We also discuss the important, but sometimes overlooked, role of aminotransferases in providing the amine group of glutamate. Finally, we discuss 5-oxoproline (5-OP, pGlu, 2-pyrrolidone-5-carboxylate, pyroglutamate) as a source of glutamate. 5-OP is of considerable interest owing to recent findings regarding the possible relationship of this compound to neurodegenerative diseases.

### 2.1. Glutaminase Versus Glutamate Dehydrogenase

Glutamate is present in plasma and could serve as a possible source of cerebral glutamate. However, in classical experiments carried out in the 1970s, Oldendorf and colleagues showed that the rat BBB possesses a low capacity system for the transport of glutamate from blood to brain [10]. This low capacity system is therefore unlikely to sustain the high levels of glutamate in normal brain. Moreover, exclusion of most of the circulating glutamate at the BBB minimizes the possibility of glutamate excitotoxicity. Thus, most of the cerebral glutamate is synthesized endogenously. The carbon skeleton of glutamate (and glutamine) is largely obtained from tricarboxylic acid (TCA) cycle-derived α-ketoglutarate and/or anaplerosis and possibly to a lesser extent from the catabolism of histidine, arginine, proline, 5-OP, and GSH.

Glutamine is a major source of brain glutamate via the glutaminase reaction: Equation (1) and reviewed in [11]. Another possible source of cerebral glutamate is the GDH reaction which catalyzes the reversible reductive amination of α-ketoglutarate: Equation (2). *In vitro* GDH can utilize either NADH or NADPH as reductant. The brain contains a considerable amount of GDH although it is somewhat heterogeneously distributed [12]. Although the forward direction (*i.e.*, in the direction of glutamate formation) is thermodynamically favorable at physiological pH values (pH 7.2–7.4) the continuous removal of ammonia by the glutamine synthetase reaction: Equation (3) ensures that the net direction *in vivo* is in the direction of glutamate oxidation to α-ketoglutarate (*i.e.*, the backward direction of Equation (2)) [13,14,15,16]. The GDH reaction will be discussed in more detail in Section 3.2.

Glutaminase: L-Glutamine + H_2_O → L-glutamate + NH_3_ (1)

GDH: α-Ketoglutarate + NH_4_^+^ + NAD(P)H ⇆ L-glutamate + NAD(P)^+^ + H_2_O (2)

Glutamine synthetase: L-Glutamate + NH_3_ + ATP ⇆ L-glutamine + ADP + P_i_ (3)

Oldendorf and Szabo demonstrated that the rat brain possesses an active transport system for the uptake of neutral amino acids [10]. Especially strong uptake was noted for the branched-chain amino acids [10]. Felig and colleagues noted that several amino acids are taken up by the human brain including the branched-chain amino acids [17]. Thus, these amino acids are a likely source of the amino group of brain glutamate. How does this occur? To answer this question we begin with a historical detour.

### 2.2. α-Ketoglutarate/Glutamate-Linked Aminotransferases

In 1957, Braunstein (the discoverer of aminotransferases (transaminases)) pointed out that coupling of an aminotransferase reaction: Equation (4) to the GDH reaction: Equation (5), shown as the reverse direction of Equation (2), provides a means of generating ammonia or of incorporating ammonia into several of the commonly-occurring amino acids: Equation (6). Braunstein coined the words “transdeamination” and “transreamination” for the forward and reverse directions of Equation (6) [18]. (For a review see [19]). These terms are rarely used nowadays, but in our opinion they serve a useful purpose because they indicate how nature has exquisitely evolved a system for shuttling nitrogen among ammonia and various amino acids as dictated by the needs of the cell [20]. However, because as noted above, the GDH reaction is a net source of ammonia in the brain the transreamination reaction does not occur in this organ to any appreciable extent under normoammonemic conditions or even under hyperammonemic conditions [14,21]. Nevertheless, under extreme hyperammonemic conditions resulting from inhibition of glutamine synthetase some cerebral ammonia may be incorporated into alanine by the transreamination route (back direction of Equation (6)), where the α-keto acid substrate is pyruvate [22].

α-Ketoglutarate-linked aminotransferase: L-Amino acid + α-ketoglutarate ⇆α-keto acid + L-glutamate (4)

GDH: L-Glutamate + NAD(P)^+^ + H_2_O ⇆ α-ketoglutarate + NH_4_^+^ + NAD(P)H (5)

***Net***: L-Amino acid + NAD(P)^+^ + H_2_O ⇆ α-keto acid + NH_4_^+^ + NAD(P)H (6)

The brain has relatively high branched-chain aminotransferase (BCAT) activity: Equation (7) and possesses *both* mitochondrial (BCAT_m_) and cytosolic (BCAT_c_) isozymes [23]. Indeed, evidence has recently been presented that BCAT_c_ in nerve endings supplies ~30% of the nitrogen for *de novo* glutamate synthesis in human brain [24]. We will come back to this point later when discussing nitrogen shuttles between astrocytes and neurons (Section 8). The salient point we wish to make here is the importance of linked aminotransferases coupled to the GDH reaction in maintaining glutamate nitrogen levels, while at the same time the carbon skeleton of glutamate is derived from TCA cycle-derived α-ketoglutarate.

Branched-chain aminotransferase: Branched-chain L-amino acid + α-ketoglutarate ⇆ branched-chain α-keto acid + L-glutamate (7)

### 2.3. Oxoprolinase

Another source of cerebral glutamate is the 5-oxoprolinase reaction: Equation (8) [25,26]. Glutamine is well known to slowly, non-enzymatically cyclize under physiological conditions to 5-OP with the elimination of ammonia. 5-OP may also be formed in the brain by the action of γ-glutamyl cyclotransferase [27] or γ-glutamylamine cyclotransferase [28] on γ-glutamyl- and γ-glutamylamine compounds, respectively. Another source of cerebral 5-OP is that derived from the hydrolysis of 5-OP-containing neuropeptides (e.g., thyrotropin releasing hormone) [29]. Baseline levels of 5-OP in the mouse brain have been reported to be ~59 nmol/g wet weight (~75 μM) [27]. The concentration of 5-OP in normal human CSF has been reported to be in the range of 10–75 μM [30,31].

5-Oxoprolinase: 5-Oxoproline + ATP + 2H_2_O → L-glutamate + ADP + P_i_ (8)

We suggest that, although 5-oxoproline is probably quantitatively a minor source of cerebral glutamate, this metabolite should not be neglected by neurochemists, especially in studies of neural cells in culture medium containing glutamine. Moreover, 5-OP should be of interest to neurochemists as a result of recent findings relating to Alzheimer disease (AD). A portion of Aβ peptide—a contributor to amyloid formation in the brain and a probable neurotoxin contributing to AD pathology—has recently been found to be truncated and to contain an N terminal 5-OP [32,33]. The full-length Aβ and the truncated forms have been designated fl-Aβ and pGlu-Aβ(3-40/42), respectively [32]. The Aβ(3-40/42) form may be especially pernicious [32]. Interventions to prevent the formation of pGlu-Aβ(3-40/42) may potentially be of benefit in the treatment of AD [32].

## 3. Major Routes for the Metabolism of Glutamate in the Brain

Major routes for the metabolism of glutamate in brain involve (1) the glutamine synthetase reaction and (2) glutamate/α-ketoglutarate-linked aminotransferases coupled to the GDH reaction. As we discuss in this section, these enzymes provide a means of eliminating excess amino acid nitrogen from the brain in the form of glutamine. Another route for the metabolism of glutamate that we briefly discuss here involves decarboxylation to the inhibitory neurotransmitter GABA. We also include in this section a brief discussion of GSH. Although the turnover of GSH in the brain is relatively slow this tripeptide is a major cerebral antioxidant and at a concentration of ~1–3 mM represents a major pool of peptide bound glutamate in the brain.

### 3.1. Glutamine Synthetase

A major route for the removal of glutamate in the brain is the glutamine synthetase reaction: Equation (3). Since glutamine is a precursor of glutamate one could consider that glutamine is a storage form of glutamate. Glutamine synthetase requires a divalent cation (Mg^2+^, Mn^2+^ or Co^2+^) for full activity. Levintow and Meister showed that in a reaction mixture containing purified glutamine synthetase, divalent cation, 10 mM ATP, 10 mM L-glutamate and 10 mM ammonia, equilibrium is attained when ~90% of the L-glutamate is converted to L-glutamine [34]. Given that the amidation of glutamate is driven by the hydrolysis of ATP it is perhaps somewhat surprising that the glutamine synthetase reaction is freely reversible *in vitro*. However, inasmuch as the concentration of ammonia in the brain (~0.18 mM; [13]) is much lower than 10 mM it is likely that the glutamine synthetase reaction in the brain *in vivo* is largely irreversible (see also Section 4.3).

The human genome contains a single gene (*GLUL*) that codes for glutamine synthetase and four *GLUL*-like genes, one of which is clearly a pseudogene [35]. Two forms of glutamine synthetase have been isolated from human brain [36,37]. One protein has a molecular weight of ~440,000 whereas the other (a glutamine synthetase-like protein) has a molecular weight of ~540,000 [36,37]. In addition to catalyzing a synthesis reaction: Equation (3), glutamine synthetase catalyzes a transferase reaction in the presence of glutamine, hydroxylamine, a divalent cation and phosphate (or arsenate): Equation (9) (ref. [38] and references cited therein). Human brain glutamine synthetase also catalyzes this transferase reaction [36]. Interestingly, the human glutamine synthetase-like protein catalyzes the synthetase reaction, but less effectively than does the glutamine synthetase enzyme [36]. On the other hand, under optimal conditions in the presence of Mn^2+^ the human glutamine synthetase-like enzyme catalyzes the transferase reaction: Equation (9) several times more rapidly than does the glutamine synthetase enzyme [36]. Boksha *et al.* [36] presented evidence that the glutamine synthetase-like protein is present in the crude mitochondrial fraction of human brain.

Glutamine synthetase (transferase reaction): L-Glutamine + NH_2_OH → L-γ-glutamylhydroxamate + NH_3_ (9)

Canine brain contains two forms of glutamine synthetase that exhibit somewhat different enzymatic properties and sensitivity to inhibition by L-methionine-*S*,*R*-sulfoximine (MSO) [39]. However, in this case, the larger form is a splice variant [39]. The human glutamine synthetase enzyme is a decamer of identical subunits. Each subunit consists of 373 amino acids. Five subunits are arranged in a circular manner over another five subunits. (See ref. [40] and references cited therein for a discussion of the architecture and some catalytic properties of human glutamine synthetase).

In the rat brain glutamine synthetase is confined mostly to astrocytes, especially in the perivascular end feet and areas surrounding synapses [41,42]. The cellular compartmentation of glutamine synthetase in astrocytes is important for the removal of neurotransmitter glutamate and the recycling of 5-carbon units back to the neurons in the form of glutamine via the brain glutamine cycle. However, at this point it is worth pointing out that certain neurons, depending on species/location/disease state, may possess some glutamine synthetase. For example, careful immunohistochemical analysis by Bernstein *et al.* [43] provided evidence that, in addition to astrocytes, subpopulations of oligodendrocytes, microglial cells and neurons in human and mouse brain express glutamine synthetase. Interestingly, the population of neurons that was shown to be immunopositive for glutamine synthetase was also shown to be immunopositive for nitric oxide synthase [43]. Moreover, immunohistochemical studies have shown that glutamine synthetase is present in a subpopulation of pyramidal neurons in AD brain but not in normal human brain [44]. The presence of neuronal glutamine synthetase is more pronounced in AD brain regions where astrocytic glutamine synthetase is depleted, particularly in the vicinity of plaques [44,45].

### 3.2. α-Ketoglutarate/Glutamate-Linked Aminotransferases Coupled to the GDH Reaction

In this section we emphasize the central importance of aminotransferases, especially AspAT (or more precisely mitAspAT): Equation (10) coupled to GDH (a mostly mitochondrial enzyme) in brain energy metabolism and glutamate homeostasis. Very high activities of both the mitochondrial (mit) and cytosolic (cyt) isozymes of AspAT are present in brain [46]. These enzymes are important components of the malate-aspartate shuttle required for the transport of reducing equivalents from cytosol to mitochondrion in lieu of NADH generated during aerobic glycolysis, e.g., [47,48].

AspAT: L-Glutamate + oxaloacetate ⇆ L-aspartate + α-ketoglutarate (10)

The AspAT reaction is so active *in vivo* that the components of Equation (10) are thought to be in thermodynamic equilibrium in many tissues as has been demonstrated for rat liver [49,50] and rat brain [51]. ^15^N-Tracer studies have also suggested that the components of the reaction are near equilibrium in synaptosomes and astrocytes [52]. The rapidity of the AspAT-catalyzed reaction *in vivo* is exemplified by the fact that administration of [^13^N]ammonia to anesthetized rats via the portal vein resulted in extremely rapid (within seconds) labeling of *both* glutamate and aspartate in the liver via the consecutive action of GDH and the two isozymes of AspAT [53]. It was demonstrated that once glutamate was labeled in the cytosol aspartate was almost instantly labeled. Equilibrium of label between aspartate and glutamate in both cytosolic and mitochondrial compartments of the rat liver was attained within minutes [53].

Previous work has shown that although most of the label derived from [^13^N]ammonia on entering the rat brain via a carotid artery cannula is incorporated into the amide position of glutamine a small amount is incorporated into glutamate [21]. Labeling of glutamate is accompanied by very rapid labeling of aspartate [21]. As a result of their high inherent activity the AspAT isozymes (mitAspAT and cytAspAT) must have very low metabolic rate coefficients: *cf*. [54] in pathways related to glutamate turnover. Thus, the enzymes will exert little metabolic control in these pathways and will act largely as passive conduits directing the flow of nitrogen and carbon as dictated by less active ancillary enzymes and the needs of the cell [20].

As noted above, there is a net uptake of certain amino acids across the BBB, especially the branched-chain amino acids. In order to maintain nitrogen homeostasis equivalent amounts of nitrogen must exit the brain. As mentioned in Section 2.2, α-ketoglutarate/glutamate-linked aminotransferases can be coupled to the GDH reaction generating ammonia from the transaminated amino acid (transdeamination: Equation (6)). This ammonia can then be incorporated into glutamine: Equation (3). A net output of glutamine to the extracellular fluid and to the circulation contributes to the maintenance of nitrogen balance in the brain (reviewed in refs. [13,55]). The flow of nitrogen is depicted in Equation (11). This pathway represents an elegant means by the brain of removing excess nitrogen from certain amino acids (e.g., branched-chain amino acids).

Flow of intracellular amino acid nitrogen to extracellular glutamine: L-Amino acid (intracellular) ⇆L-glutamate ⇆ ammonia → L-glutamine (intracellular) → L-glutamine (extracellular) (11)

Interestingly, humans possess two GDH isozymes, namely hGDH1 and hGDH2 [56]. GDH1 is common to all mammals, whereas GDH2 arose by gene duplication in the higher primates (ref. [56] and references cited therein). Among human tissues GDH2 is especially prominent in brain. As pointed out by Spanaki *et al.* [56] the evolution of hGDH2 was driven by the bestowal to this enzyme of enhanced catalytic ability under conditions that are inhibitory to the ancestral enzyme. Immunohistochemical studies of human cortex revealed that hGDH1 is expressed in glial cells, including astrocytes, but not in neuronal cells. On the other hand, immunohistochemical studies revealed the presence of hGDH2 in both astrocytes and neurons [56]. Staining was noted to be especially prominent in the presynaptic nerve terminals of the large human neurons [56]. According to Spanaki *et al.*, “hGDH2 evolution bestowed large human neurons with enhanced glutamate metabolizing capacity, thus strengthening cortical excitatory transmission” [56].

Why does the brain contain high levels of GDH? As noted above GDH linked to aminotransferases facilitates the flux of excess amino acid nitrogen toward ammonia and glutamine (amide). However, there is yet another important role for cerebral GDH. Consider, for example, steroidogenesis. Steroid-producing cells (e.g., cells of the adrenal cortex) contain substantial amounts of both hGDH1 and hGDH2 [57]. Conversion of acetyl-CoA to cholesterol requires the oxidation of many molar equivalents of NADPH to NADP^+^. As noted above, GDH can utilize either NAD^+^ or NADP^+^ as cofactor. However, because the concentration of NADPH is greater than that of NADH in most cells NADPH is generally utilized in preference over NADH in biosynthetic reductions. Thus, it is perhaps not surprising that the GDH reaction acts as a convenient source of NADPH: Equation (5) required for steroidogenesis. The role of steroids in the brain was a long neglected area of research, but recently neurosteroids (*i.e.*, steroids generated from cholesterol in the brain) have become an intense area of research. For example, some neurosteroids have been shown to (1) be neuroprotective; (2) directly modulate the channel properties of synaptic neurotransmitter receptors; and (3) regulate neural function, behavior, and cognition (reviewed in ref. [58]). Thus, the GDH reaction, not only functions to direct excess amino acid nitrogen toward ammonia in the brain, but at the same time it also serves as a source of NADPH for important biosynthetic reactions, including steroidogenesis. This is an excellent example of a case where nature has parsimoniously “engineered” a seemingly simple reaction to serve two disparate, but extremely important functions.

### 3.3. Ammonia Generated by Aminotransferase Reactions Coupled to the Purine Nucleotide Cycle

The purine nucleotide cycle (PNC) was described by Lowenstein in the 1960s [59]. The PNC operates by the consecutive action of three enzymes: adenylosuccinate synthetase: Equation (12), adenylosuccinate lyase: Equation (13), and AMP deaminase: Equation (14). The net reaction is shown in Equation (15). Work by Schultz and Lowenstein has established that the PNC is present in brain [60,61]. These authors suggested that the PNC is a major source of ammonia in the brain. To our knowledge the proposed role of the PNC in brain nitrogen and energy metabolism has been little investigated since the seminal publications by Schultz and Lowenstein in the mid-1970s. One report suggests that nerve terminals have a limited PNC [52]. However, loss of adenylosuccinate lyase activity is associated with severe neurological problems, e.g., [62], suggesting an important metabolic role of this enzyme in the brain. Moreover, immunohistochemical analysis and *in situ* hybridization showed relatively high levels of AMP deaminase protein and message in rat brain neurons [63]. In our opinion the importance of the PNC in brain nitrogen and energy metabolism is a neglected area of research.

Adenylosuccinate synthetase: Inosine monophosphate (IMP) + L-aspartate + GTP → adenylosuccinate + GDP + P_i_ (12)

Adenylosuccinate lyase: Adenylosuccinate → AMP + fumarate (13)

AMP deaminase: AMP + H_2_O → IMP + NH_3_ (14)

***Net***: L-Aspartate + GTP + H_2_O → fumarate + GDP + P_i_ + NH_3_ (15)

By linking an α-ketoglutarate-dependent aminotransferase: Equation (4) (e.g., branched-chain aminotransferase) to the AspAT reaction: Equation (10) and the PNC: Equation (15) the amino acid nitrogen is incorporated into ammonia and thence into the amide position of glutamine: Equation (16). An advantage of this pathway is that coupling of ammonia production to GTP hydrolysis makes the pathway energetically favorable.

Flow of intracellular amino acid nitrogen to glutamine amide via the PNC: L-Amino acid (intracellular) ⇆ L-glutamate ⇆ L-aspartate → ammonia → L-glutamine (intracellular) → L-glutamine (extracellular) (16)

Lowenstein and Goodman [64] have pointed out that coupling of the PNC to an α-ketoglutarate/glutamate-linked aminotransferase, AspAT, fumarase and malate dehydrogenase can drive the deamination of an amino acid. The net result of these coupled reactions is shown in Equation (17). Note that owing to the hydrolysis of GTP the overall reaction shown in Equation (17) is more favorable for the deamination of an amino acid than is the transdeamination pathway: Equation (6). Additionally, if Equation (4) is omitted from the sequence then this results in a deamination of glutamate: Equation (18) that is energetically more favorable than that obtained by the GDH reaction: Equation (5). For a more detailed discussion of these pathways see ref. [20].

Deamination pathway for an amino acid linked to the PNC (general): L-Amino acid + NAD(P)^+^ + GTP + 2H_2_O → α-keto acid + NH_4_^+^ + NAD(P) H + GDP + P_i_ (17)

Deamination pathway for glutamate linked to the PNC: L-Glutamate + NAD(P)^+^ + GTP + 2H_2_O → α-ketoglutarate + NH_4_^+^ + NAD(P)H + GDP + P_i_ (18)

The importance of the AspAT isozymes in energy metabolism is further underscored by the fact that the two α-keto acids involved in the transamination reaction—α-ketoglutarate and oxaloacetate—are entry points into the TCA cycle. Thus, glutamate and aspartate carbon are interconvertible to a considerable extent in many tissues. By linking mitAspAT to certain enzymes of the TCA cycle it is possible to construct two routes by which aspartate is converted to glutamate. The net reactions are shown in Equations (19) and (20). On the other hand, by linking mitAspAT to the bottom half of the TCA cycle (as usually depicted) it is possible to construct a route for the net conversion of glutamate to aspartate: Equation (21). The conversion of glutamate carbon to aspartate carbon in liver mitochondria was first recognized more than 65 years ago and under certain circumstances may be almost quantitative [65]. Note that the conversion of α-ketoglutarate to oxaloacetate yields a considerable amount of energy (~9 high energy phosphate bonds). Because the AspAT reaction is freely reversible (*K’* ~7 for the direction shown in Equation (10)) [66] this will have little impact on energy released by net oxidation of glutamate to aspartate *versus* that released by oxidation of α-ketoglutarate to oxaloacetate. Thus, conversion of glutamate to aspartate, which is in effect a truncated TCA cycle, will also yield a similar amount of energy. For more complete descriptions of the interconversion of glutamate and aspartate see ref. [67] and [68].

Conversion of aspartate carbon to glutamate carbon via the TCA cycle (1): L-Aspartate + acetyl-CoA + ½O_2_ → L-glutamate + CO_2_ + CoA (19)

Conversion of aspartate carbon to glutamate carbon via the TCA cycle (2): 2L-Aspartate + 1½O_2_ → L-glutamate + NH_3_ + 3CO_2_ + H_2_O (20)

Conversion of glutamate carbon to aspartate carbon via the TCA cycle: L-Glutamate + 1½O_2_ → L-aspartate + CO_2_ + H_2_O (21)

The equations discussed above serve to emphasize the interrelatedness of nitrogen and energy metabolism in the brain and the pivotal role of linked aminotransferases (including AspAT) in this process. *Notably, these equations highlight the importance of glutamate as a keystone metabolite in cerebral nitrogen homeostasis*.

### 3.4. Conversion of L-Glutamate to GABA

The brain contains a considerable amount of glutamate decarboxylase which catalyzes the decarboxylation of L-glutamate to GABA: Equation (22). The concentration of GABA (the major inhibitory neurotransmitter) in the brain is ~0.5–3 mM depending on the species (Table 1). Since the carbon skeleton of GABA is derived from α-ketoglutarate, formation of GABA represents possible loss of carbon from the cerebral TCA cycle. However, the four carbons of GABA are reincorporated into the TCA cycle via the GABA shunt (also known as the GABA bypath) which consists of a GABA aminotransferase: Equation (23) and succinic semialdehyde dehydrogenase: Equation (24). The succinate thus formed enters the TCA cycle and the electrons associated with NADH enter the electron transport chain. The net reaction is shown in Equation (25). The GABA shunt was first elucidated in the 1960s and shown to be compartmentalized in the brain by Balázs and colleagues in 1970 (ref. [69] and references quoted therein). (For a recent review of the possible role of the GABA shunt/GABA metabolites in AD see ref. [70].) The GABA shunt will be discussed later in regard to the glutamine-GABA cycle.

Glutamate decarboxylase: L-Glutamate → GABA + CO_2_ (22)

GABA aminotransferase: GABA + α-ketoglutarate ⇆ succinic semialdehyde + L-glutamate (23)

Succinic semialdehyde dehydrogenase: Succinic semialdehyde + NAD^+^ → succinate + NADH (24)

***Net***: α-Ketoglutarate + NAD^+^ → succinate + NADH + H^+^ (25)

### 3.5. GSH as a Glutamate Reservoir

The turnover of GSH in the adult brain is relatively slow. For example, Douglas and Mortenson [71] injected [^14^C]glycine into adult rats, noted the time of peak appearance of label in brain GSH and then measured the rate of decline of label in this pool of GSH. From the results of this experiment the authors estimated a t_½_ of ~71 h for the turnover of GSH in rat brain [71]. Thus, the contribution of the turnover the glutamate moiety of brain GSH to the rate of turnover of the *total* brain glutamate pool is evidently relatively minor. Nevertheless, a discussion of brain GSH is included here because GSH, along with ascorbate, are the major water soluble antioxidants of the brain. Moreover, the concentration of GSH in the brain is such that this antioxidant represents a major pool of peptide-bound glutamate. The concentration of GSH in the brain ranges from ~0.25 to 3.0 mM depending on the species compared to 6–12 mM for glutamate (Table 1) [72,73].

GSH is synthesized in two ATP-dependent steps. The first step is catalyzed by γ-glutamylcysteine ligase: Equation (26); formerly known as γ-glutamylcysteine synthetase. The second step is catalyzed by glutathione synthetase: Equation (27). The first enzyme-catalyzed step is generally considered as rate controlling. However, recent work suggests that the uptake of cysteine is the greater determinant of GSH production in brain [74]. In astrocytes the uptake of cysteine is mediated by the excitatory amino acid transporter 3 (EAAT-3) and the ASC system that transports alanine, serine, and cysteine [74]. GSH is more prevalent in astrocytes than in neurons [75,76]. A significant portion of the astrocytic GSH pool is released to the interstitial space where it is hydrolyzed to γ-glutamylcysteine and glycine by the action of the ectoenzyme γ-glutamyltransferase located on the exofacial leaflet of the astrocyte plasmalemma. The peptide linkage of γ-glutamylcysteine is, in turn, hydrolyzed by a neuronal ectopeptidase to yield glutamate and cysteine that are actively transported into neurons by EAAT-3 and EAAT-2, and thereby, contribute to GSH synthesis in these cells [74,77,78,79].

At least five Na^+^-dependent glutamate transporter subtypes have been identified in neural tissues (ref. [80] and references cited therein). These include GLAST (glutamate aspartate transporter, EAAT-1) and GLT-1 (glutamate transporter-1, EAAT-2), which are primarily expressed in astrocytes [81,82]. Thus, although some glutamate is taken up into neurons via the EAAT-3 transporter, the bulk of extracellular glutamate released as a result of neurotransmission is taken up by glutamate transporters into astrocytes. These transporters are important components of the glutamine cycle (Section 6).

Interestingly, the wild type prion protein (PrP) binds to EAAT-3 and enhances the uptake of glutamate and cysteine by neuronal EAAT-3 [74]. PrP also associates with, and increases the activity of, γ-glutamyl transferase [74]. These observations suggest that wild-type PrP regulates GSH synthesis in the astrocyte-neuron circuit by promoting specific protein-protein interactions that favor the production of this tripeptide in neurons [74]. Wild-type PrP is expressed throughout the body but is most abundant in the central nervous system (CNS) where it is thought to be neuroprotective (ref. [74] and references quoted therein). Wild-type PrP functions as a sensor for oxidative stress [83,84] and triggers intracellular signal transduction cascades that act on antioxidant systems, such as the GSH system [74].

γ-Glutamylcysteine ligase: L-Glutamate + L-cysteine + ATP → L-γ-glutamylcysteine + ADP + P_i_ (26)

Glutathione synthetase: L-γ-Glutamylcysteine + glycine + ATP → GSH + ADP + P_i_ (27)

Given the central role of GSH as a major water soluble antioxidant it would be greatly advantageous to be able to measure cerebral GSH *in vivo* under normal and pathological conditions by, for example, proton magnetic resonance (MR) spectroscopy. However, detection and quantitation of brain GSH *in vivo* by proton MR spectroscopy is challenging owing to the fact that peaks assigned to GSH overlap with those assigned to glutamate and glutamine. Nevertheless, as the sensitivity of MR techniques continues to improve *in vivo* proton MR spectroscopy can now be applied to quantitative measurements of glutamate, glutamine and GSH in rodent brain and more recently in human brain (see ref. [85] and references cited therein). This technique will be especially relevant in measuring changes of human brain GSH under normal and pathological conditions [85].

## 4. The Glutamate Buffer in the Brain

### 4.1. Glutamate as a Nitrogen Buffer in the Brain

**Figure 2:**
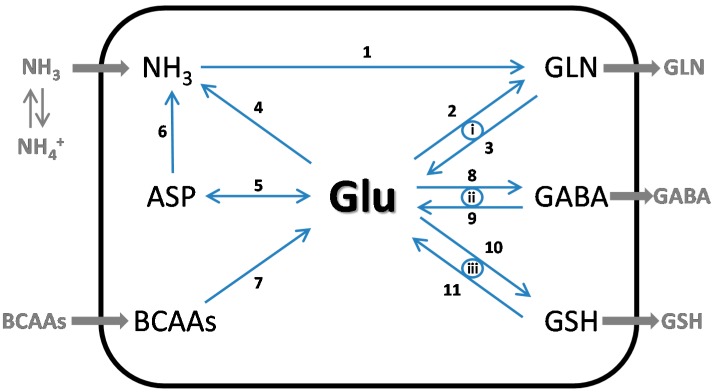
One-compartment model highlighting the central role of glutamate as a nitrogen buffer/bulwark in the brain. The diagram is greatly simplified to omit cellular and subcellular compartmentation of enzymes involved in maintaining nitrogen homeostasis. The gray arrows (**left**) indicate the major flow of nitrogen from blood to cells and then to interstitial fluids (**right**) irrespective of compartmentation. For simplicity other sources of nitrogen such as those involved in the transamination of glutamine and in purine and pyrimidine catabolism are omitted. Moreover, turnover of compounds in the brain associate with these pathways is likely to be relatively slow compared to that of glutamate. Ammonia enters the brain largely by diffusion of NH_3_. Additional nitrogen enters the brain via transport of amino acids, especially the branched-chain amino acids (BCAAs). **Enzymes**: **1**, Glutamine synthetase (ammonia as substrate); **2**, glutamine synthetase (glutamate as substrate); **3**, glutaminase; **4**, glutamate dehydrogenase; **5**, mit and cyt aspartate aminotransferases; **6**, enzymes of the purine nucleotide cycle; **7**, branched-chain amino acid aminotransferases; **8**, glutamate decarboxylase; **9**, enzymes of the GABA shunt; **10**, glutamate cysteine ligase + glutathione synthetase; **11**, γ-glutamyltransferase + cysteinylglycine dipeptidase. **Cycles between astrocytes and neurons**: **i**, Glutamine cycle; **ii**, glutamine-GABA cycle; **iii**, the three amino acid components of glutathione synthesized in the astrocytes are recycled and assembled anew in the neurons.

From the above discussion it is apparent that glutamate is a key buffer/bulwark in mechanisms by which the brain maintains nitrogen homeostasis. This is exemplified by tracing major sources of nitrogen input (e.g., ammonia, branched-chain amino acids) and output (e.g., glutamine) as shown in Figure 2.

### 4.2. Glutamate as an Energy Buffer in the Brain

**Figure 3:**
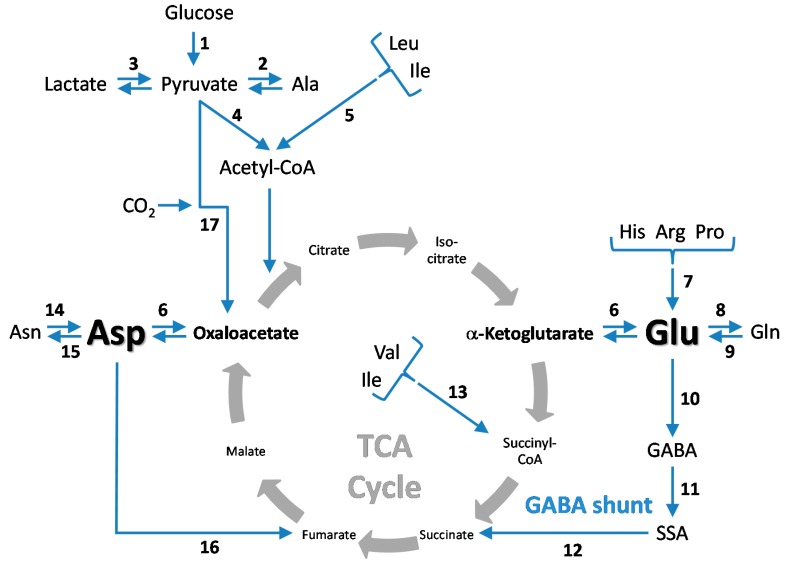
One-compartment model highlighting the central role of glutamate as a buffer of **C4/C5** **intermediates** in the brain. The diagram shows the standard depictions of the TCA cycle and GABA shunt in the brain. In addition the diagram indicates how glutamate acts as a buffer for C5 intermediates (α-ketoglutarate, glutamine) and C4 intermediates (GABA, succinic semialdehyde (SSA), succinate, fumarate, oxaloacetate, aspartate, and asparagine). **Key enzymes/metabolic pathways**: **1**, Aerobic glycolysis; **2**, alanine aminotransferase; **3**, lactate dehydrogenase; **4**, pyruvate dehydrogenase complex; **5**, metabolism of leucine and isoleucine (in part) to acetyl-CoA; **6**, aspartate aminotransferase; **7**, metabolism of histidine, arginine and proline resulting in the incorporation of 5-carbon units into glutamate; **8**, glutamine synthetase; **9**, glutaminase; **10**, glutamate decarboxylase; **11**, GABA aminotransferase; **12**, succinic semialdehyde dehydrogenase; **13**, anaplerotic metabolism of isoleucine (in part) and valine to succinyl-CoA; **14**, asparagine synthetase; **15**, asparaginase; **16**, enzymes of the purine nucleotide cycle; **17**, anaplerotic pyruvate carboxylase. Note that the pentose phosphate pathway is present in brain but is not included in this diagram. Also not shown are all the reaction intermediates and cofactors.

Glutamate is not only a key figure in brain nitrogen homeostasis (Figure 2), but it is also a key figure in the maintenance of 5-C and 4-C intermediates in brain energy metabolism (Figure 3). A crucial step in this process is the mitAspAT-catalyzed rapid shuttling of carbon between glutamate and α-ketoglutarate of the TCA cycle. Indeed, this step is so active that α-ketoglutarate and glutamate and can be treated as a single combined kinetic pool in MR modeling of the *in vivo* TCA cycle flux in the rat brain [86]. By using sophisticated MR techniques Mason *et al.* estimate that exchange between α-ketoglutarate carbon and glutamate carbon in the rodent brain is ~100 times faster than is flux through the TCA cycle [86]. These authors calculated that TCA flux in the rodent brain is ~1.58 μmol/min/g [86]. Estimates of TCA flux using MR technique in the neocortices of healthy, adult volunteers is ~0.37 μmol/min/g [86]. Recent estimates of carbon flux in the anesthetized mouse brain using MR techniques indicate a flux through the anesthetized mouse brain of ~1.05 μmol/min/g [87]. This value is slightly higher than estimates for TCA flux in human and rat brain (reviewed in ref. [87]). In the study of Xin *et al.*, the rate of exchange between mitochondrial α-ketoglutarate and cytosolic glutamate was found to be ~0.48 μmol/min/g—a value of the same magnitude as that of the TCA cycle [88]. This finding is reminiscent of our previous findings (noted above) of rapid equilibration of the components of the AspAT reaction in rat liver.

### 4.3. Cerebral Glutamate Buffering during Hyperammonemia

Hyperammonemia occurs in many diseases including urea cycle defects and liver disease (reviewed in ref. [13]). The *K*_m_ value for ammonia reported for purified sheep brain glutamine synthetase is 180 μM [89]. Best estimates for the concentration of ammonia in the normal rat whole brain are ~180 μM [13]. However, the concentration may be even less in the glutamine synthetase-rich astrocytes. Thus, it is likely that mammalian brain glutamine synthetase is not saturated with ammonia under normoammonemic conditions. It is important to note that (1) ammonia enters the brain largely by diffusion [90]; and (2) the major route for glutamine metabolism is by the glutaminase reaction which occurs predominantly in a compartment separate from that of the astrocytes (*i.e.*, neurons; see below). A small decrease in the specific activity of cerebral glutamine synthetase has been reported for hyperammonemic, portacaval shunted rats [21,91] and for hyperammonemic liver disease patients [92]. On the other hand, an increase in the specific activity of cortex and cerebellum glutamine synthetase has been reported for hyperammonemic rats with acute liver failure [93]. Overall, it is likely that hyperammonemia will lead to an increased rate of glutamine synthesis and increased glutamine concentration in the brain. Indeed, it has long been known that hyperammonemia in experimental animal leads to increased brain glutamine concentrations [13,94,95,96]. The concentration of glutamine is also increased in biopsied brain tissue from patients dying of liver disease [92]. MR experiments have also demonstrated large increases *in vivo* in the concentration of brain glutamine in hyperammonemic rats [97] and in hyperammonemic patients with liver disease [98,99].

Given that the concentration of brain glutamine greatly increases during hyperammonemia and that glutamine is synthesized directly from glutamate one might expect that hyperammonemia will result in a large decrease in brain glutamate. Certainly, brain glutamate levels are decreased in animal models of hyperammonemia [94,95,96,97], and in hyperammonemic cirrhotics [98,99]. However, the decrease in glutamate concentration is much less than the increase in glutamine concentration. Moreover, the concentration of the major 5-carbon precursor of both glutamate and glutamine (*i.e.*, α-ketoglutarate) in the brain is increased in chronically hyperammonemic mice with a defect in ornithine transcarbamylase [100]. Increased α-ketoglutarate levels may be related in part to a possible ammonia-induced inhibition of the α-ketoglutarate dehydrogenase complex [101]. Previously we reported concentration values for brain α-ketoglutarate, glutamate, and glutamine in four groups of rats: (1) rats infused with sodium acetate (controls); (2) rats infused with ammonium acetate; (3) rats pretreated with MSO (a potent glutamine synthetase inhibitor) followed by infusion with sodium acetate, and (4) rats pretreated with MSO followed by infusion with sodium acetate [102]. The findings are summarized in Table 2. The table shows that infusion of ammonium acetate or inhibition of brain glutamine synthetase by prior administration of MSO results in marked hyperammonemia. The hyperammonemia is most pronounced when the rats are pretreated with MSO and then infused with ammonium acetate. Interestingly, however, the concentration of α-ketoglutarate is either not changed (in ammonium acetate-treated rats) or is moderately, but significantly elevated (in MSO/sodium acetate-treated rats and in MSO/ammonium acetate treated rats) (Table 2). These findings provide additional evidence to that mentioned above that the cerebral GDH reaction does not proceed in the direction of glutamate formation, but rather in the direction of glutamate oxidation. Of considerable interest is the very modest decrease in brain glutamate in the rats treated with ammonium acetate but very large increase in brain glutamine. This finding emphasizes the fact that the total amount of TCA cycle-derived 5-carbon compounds (*i.e.*, α-ketoglutarate plus glutamate plus glutamine) is greatly elevated in the hyperammonemic rat brain in the presence of uninhibited glutamine synthetase (Table 2). How is this possible? One possibility is that anaplerosis resulting from the metabolism of branched-chain amino acids (especially isoleucine) contributes to the α-ketoglutarate precursor necessary for the synthesis of hyperammonemia-induced increases in the concentration of glutamate and glutamine [103]. However, this contribution is likely to be modest [103]. A major route for anaplerosis in the brain appears to be CO_2_ fixation and this is discussed in the next section.

## 5. CO_2_ Fixation in the Brain—Stimulation by Hyperammonemia

### 5.1. Cerebral CO_2_ Fixation during Normoammonemia

The brain possesses four enzymes that have the potential to fix CO_2_, namely pyruvate carboxylase, malate enzyme, phosphoenolpyruvate carboxykinase, and propionyl-CoA carboxylase (ref. [104] and references cited therein). Some evidence has been presented that the malic enzyme: Equation (28) under certain conditions is responsible for some CO_2_ fixation in neurons [105,106]. However, McKenna *et al.* [107] have presented evidence that the malate enzyme in the brain is especially enriched in synaptic mitochondria where it operates in the direction of pyruvate formation. In that case, the malate enzyme rather than fixing CO_2_ acts as a net producer of CO_2_—the reverse direction of Equation (28). Under conditions of low aerobic glycolysis in the brain the malate enzyme would provide pyruvate for continuous operation of the TCA cycle in which carbon from glutamate and glutamine is directed first toward α-ketoglutarate and then to malate through the TCA cycle and finally to pyruvate [107].

Malic enzyme: Pyruvate + CO_2_ + NADPH + H^+^ ⇆ (*S*)-malate + NADP^+^ (28)

Not all glucose taken up by the brain is completely oxidized to CO_2_. A small portion (~5%) is normally released during aerobic glycolysis from the brain as lactate (ref. [108] and references cited therein). As pointed out by Sonnewald [109] anaplerosis in the brain must be exactly balanced by cataplerosis. Anaplerotic carbon entering the TCA cycle can be offset to some extent by cataplerotic loss of carbon to the CSF and circulation in the form of glutamine. This process also balances nitrogen input and export. Aerobic loss of lactate from the brain also represents a cataplerotic process [109]. This lactate is not obtained solely by reduction of pyruvate derived glycolytically from glucose. Rather much of the lactate released to the circulation originates from cataplerotic loss of CO_2_ via the conversion of malate to pyruvate catalyzed by the malate enzyme—the reverse direction of Equation (28) [109,110].

Based on the considerations in the previous paragraph it is unlikely that the malate enzyme could be a source of *increased* cerebral CO_2_ fixation during hyperammonemia. Moreover, the capacity of phosphoenolpyruvate carboxykinase and propionyl-CoA carboxylase to fix CO_2_ in the brain is limited [104]. It has been known for more than 30 years that astrocytes contain the major pool of pyruvate carboxylase: Equation (29), in the brain and that the carbon fixed by this enzyme is a source of glutamate and glutamine carbon [110,111]. As noted above, glutamine synthetase activity is also prominent in astrocytes. The co-localization of these two enzymes in astrocytes has important ramifications concerning cerebral glutamate and glutamine metabolism as noted below.

Pyruvate carboxylase: Pyruvate + CO_2_ + ATP → oxaloacetate + ADP + P_i_ (29)

In agreement with the earlier work several ^14^C labeling studies and *in vivo* MR studies have shown that the importance of the combined action of pyruvate carboxylase and glutamine synthetase for net glutamine synthesis in the brain (ref. [112] and references cited therein). For example, using a two-compartment model in awake rats and measurement of label disposition derived from ^14^C-bicarbonate and [1-^13^C]glucose it was shown that TCA cycle activity in glia (~0.5 μmol/min/g) is about 30% that of the whole brain and on a par with flux through the glutamine cycle (0.5–0.6 μmol/min/g) (discussed in Section 6 below) [113]. Moreover, the anaplerotic fixation of CO_2_ by the pyruvate carboxylase reaction in the glial compartment is remarkably high (0.14–0.18 μmol/min/g) in the awake rat [113]. The value is somewhat lower in the anesthetized mouse brain (~0.09 μmol/min/g) [114]. Nevertheless carbon flux through the glial pyruvate carboxylase reaction is substantial in these animals—calculated to be 37% relative to flux through the glutamine cycle [114]. These findings build on previous studies showing that a considerable portion of the carbon incorporated into the glutamine compartment is due to anaplerotic CO_2_ fixation (20%–35% depending on species) in humans [115,116], rats [117,118], and rabbits [119].

### 5.2. Cerebral CO_2_ Fixation during Hyperammonemia

It has long been known that hyperammonemia results in considerably increased CO_2_ fixation into amino acids in the brains of experimental animals [120,121]. Based on considerations in the previous paragraph it is probable that CO_2_ fixation accounts for much of the increase in 5-carbon compounds (α-ketoglutarate plus glutamate plus glutamine) noted in hyperammonemic brain (Table 2). As shown by the data in Table 2 most of the hyperammonemia-induced increase in 5-carbon metabolites in animal models of hyperammonemia is due to glutamine. Increased levels of cerebral glutamine have also been detected *in vivo* in hyperammonemic patients with hepatic encephalopathy [98,99,122,123]. Because hyperammonemia stimulates pyruvate carboxylase (an ATP-dependent reaction) and may inhibit α-ketoglutarate dehydrogenase complex activity it is theoretically possible that increased glutamine production will result in a cerebral energy deficit. However, the subject is somewhat controversial and although aspects of cerebral energy metabolism are clearly altered by hyperammonemia in both animal models and liver disease patients [124,125] the overall effect on brain energy metabolism is not pronounced except perhaps in the agonal state. For example, ^1^H- and ^32^P MR studies of cerebral metabolites in rats infused with ammonium acetate showed minimal changes in high energy phosphate production [126]. Similarly, ^1^H- and ^32^P MR studies in a model of chronic hepatic encephalopathy (bile duct ligation in the rat) suggest minimal interference with cerebral high energy phosphate production [127]. Nevertheless, some aspect of altered cerebral metabolism must account for the encephalopathy associated with hyperammonemia. Currently, excess glutamine production is thought to be a major culprit. This excess glutamine results in disruption of the glutamine cycle (discussed in the next section), pathological stress in astrocytes, inflammation and brain edema (reviewed in ref. [128,129]). However, the authors of a recent study of chronically hyperammonemic rats (bile duct ligation model) have suggested that overproduction of lactate rather than glutamine may be a more important factor in the production of brain edema [130]. Thus, the mechanism by which hyperammonemia results in brain edema still remains controversial.

## 6. Cerebral Glutamine Cycle

**Figure 4:**
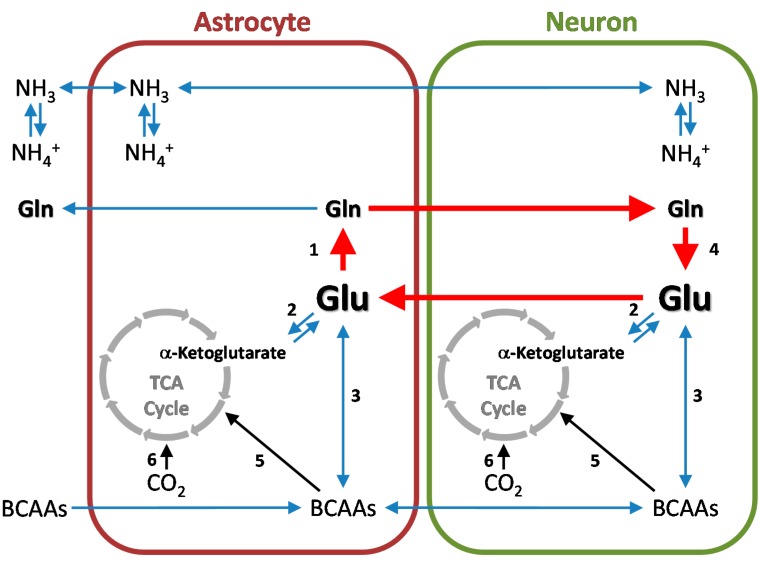
Cerebral glutamine cycle and ancillary reactions. Under normal conditions ammonia enters the brain mostly by diffusion of the free base (NH_3_) [90]. This ammonia, and that derived from endogenous reactions, is metabolized primarily via incorporation into the amide position of L-glutamine in a reaction catalyzed by astrocytic glutamine synthetase (reaction **1**). Although the GDH reaction is freely reversible, this enzyme is a net source of ammonia (reaction **2**) rather than a net remover of ammonia. The glutamate required for glutamine synthesis in the astrocytes is derived in part from glutamate released from neurons during neurotransmission; the nitrogen of this glutamate may be obtained by transamination of α-ketoglutarate with suitable amino acids (e.g., branched-chain amino acids, BCAAs) (reaction **3**). Some of the glutamine formed in the glutamine synthetase reaction may be released to the circulation to maintain nitrogen and carbon homeostasis. Another portion may be returned to the neurons, wherein it is converted back to glutamate by the action of glutaminase(s) (reaction **4**). The sequence Glu (neurons) → Glu (astrocytes) → Gln (astrocytes) → Gln (neurons) → Glu (neurons) constitutes the cerebral glutamine cycle. As discussed in the text anaplerotic reactions occur in the brain and may be used to replenish 5-C units. Such anaplerotic reactions include CO_2_ fixation by pyruvate carboxylase (→, reaction **6**) and metabolism in part of branched-chain amino acids (→, reaction **5**). For simplicity the glutamine-GABA cycle is not shown. Although ammonia produced in neurons is kinetically distinct from that produced in astrocytes, this ammonia must eventually enter the astrocytes wherein it is a substrate of glutamine synthetase. This is accomplished by diffusion of the free base (NH_3_) or by ammonium (NH_4_^+^) transporters [133].

Studies of [^15^N]ammonia metabolism in cat brain [131] and later studies of [^13^N]ammonia metabolism in rat brain [14] are consistent with metabolic compartmentation of nitrogen metabolism in rat brain. Briefly, ammonia entering the brain from the blood is rapidly converted to glutamine in a glutamate-utilizing compartment that turns over more rapidly than a larger, distinct glutamate compartment. In part through the important work of Norenberg and colleagues [41,42] the small compartment is known to be represented by astrocytes and the large compartment by neurons. Benjamin and Quastel were the first to describe an important role for this compartmentation [132]. These authors suggested that the brain contains a glutamine cycle—astrocytes take up glutamate released from neurons during transmission and release glutamine to the neurons; the neurons then accumulate glutamine as a precursor for neurotransmitter glutamate [132]. The glutamine cycle and important ancillary reactions are shown in Figure 4.

Cleary the cerebral glutamine cycle is not a closed system because as discussed above, anaplerosis and cataplerosis will add or remove carbon from the cerebral TCA cycle, respectively, which in turn will affect the flow of individual carbon atoms into and out of the glutamine cycle components. Nevertheless mass balance must be maintained. Various studies with ^14^C-labeled precursors (in rodents) and MR studies (in rodents and humans) have shown that that about 80% of cerebral glutamine synthesis is normally associated with glutamate neurotransmission and about 20% is associated with anaplerosis (reviewed in ref. [134]). ^13^C MR studies have also shown that the glutamatergic neurotransmitter flux is substantial. For example, the flux though glutamine/glutamate cycling occurs at ~30%–40% that of the neuronal TCA cycle flux in the brains of anesthetized rats and ~38%–50% in the brains of awake rats and in human cerebral cortex (reviewed in ref. [135]).

A further caveat to treating the glutamine cycle as a closed circuit is that as discussed above (Section 3.1), human and dog brain possess at least two glutamine synthetases. Moreover, although glutamine synthetase is highly enriched in astrocytes it is now apparent that some subpopulations of neurons possess glutamine synthetase and that the enzyme is aberrantly expressed in a subpopulation of neurons in AD brain (Section 3.1). One group found very low glutamine synthetase activity in rat brain synaptosomes [136]. However, at least two groups have reported the presence of appreciable glutamine synthetase activity in synaptosomes [137,138]. Glutamine synthetase in neurons may have a function distinct from the well-established role it plays in the glutamine cycle. Clearly the presence of multiple forms of glutamine synthetase in some mammalian species and the presence of glutamine synthetase activity in some neurons (and possibly in nerve endings) and in microglia (next paragraph) are important areas for further investigation.

As noted above, hyperammonemia is a major contributing factor to the neuropathology associated with acute and chronic liver failure. As also noted above, hyperammonemia results in increased cerebral glutamine concentrations. Elevated brain glutamine is associated with brain edema, especially in acute liver failure, e.g., [128,129,139]. Brain edema in liver disease patients has been demonstrated directly by MR techniques [98,123]. However, the mechanism by which excess glutamine induces brain edema is not fully understood. Brusilow and colleagues have strongly championed the hypothesis that brain edema in hepatic encephalopathy is largely due to an osmotic stress resulting from a hyperammonemia-induced increase of glutamine concentration in astrocytes (the major site for the synthesis of glutamine in the brain) [128]. Others have hypothesized that microglia and neuroinflammation play an important role in promoting the edema associated with hepatic encephalopathy. However, the two hypotheses are not mutually exclusive—increased astrocyte swelling may contribute to the neuroinflammatary process or *vice versa*. It is now recognized that hyperammonemia produces not only neuroinflammation but also a systemic inflammatory response. Trannah *et al.*, note “systemic inflammation develops following liver injury, resulting in hyperammonemia and a ‘cytotoxic soup’ of pro-inflammatory mediators which are released into the circulation and modulate the impact of ammonia on the brain” [139]. Jayakumar *et al.*, have recently summarized the inflammatory mechanisms in acute hepatic encephalopathy by which activation of endothelial cells and microglia have been suggested to impact on astrocytes, leading to their dysfunction, ultimately contributing to astrocyte swelling/brain edema [140]. Recently it was shown that primary cultures of rat microglia possess glutamine synthetase protein [141]. Moreover, cortical microglia have been shown to possess GLAST, which is upregulated by nicotine [142]. It was suggest that increased GLAST expression clears glutamate from the synapse and decreases glutamate neurotransmission [142]. In the quiescent state little conversion of extracellular glutamate to glutamine by the microglia could be detected [141]. However, when activated by lipopolysaccharide the cells exhibited lower levels of glutamine synthetase protein yet markedly increased ability to convert glutamate to glutamine [141], presumably in part due to a strong glutamate transport system. This ability of activated cerebral microglia to convert extracellular glutamate to glutamine may be stimulated by hyperammonemia due to the presence of increased substrate (*i.e.*, ammonia). The effect of increased microglial glutamine synthesis on the functioning of the cerebral glutamine cycle during liver disease/hyperammonemia is unknown. We suggest that increased conversion of extracellular glutamate to glutamine in activated microglia may interfere with glutamate transmission thereby contributing in part to hyperammonemia-induced encephalopathy.

## 7. Cerebral Glutamine-GABA Cycle

**Figure 5:**
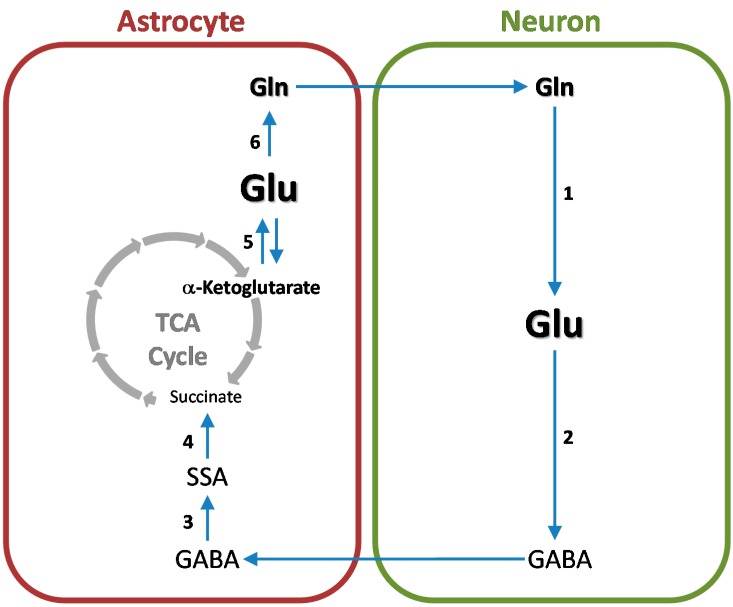
Flow of carbon through the cerebral glutamine-GABA cycle. For simplicity the glutamine cycle, GABA/glutamine transporters and anaplerotic reactions are omitted. **Enzymes/metabolic pathways**: **1**, Glutaminase; **2**, glutamate decarboxylase; **3**, GABA aminotransferase; **4**, succinic semialdehyde (SSA) dehydrogenase; **5**, α-ketoglutarate-linked aminotransferases; **6**, glutamine synthetase.

GABAergic neurons are abundant in the mammalian brain. For example, in the cat striatal cortex 11% of synapses are GABA-immunopositive and therefore originate from GABAergic neurons [143]. Once GABA is released from GABAergic neurons it is taken up mostly by astrocytes wherein it is converted to glutamine. Glutamine is then returned to the neurons wherein it is converted to GABA thus completing the glutamine-GABA cycle. (For a recent review see ref. [144]). The basic outline of the cycle is depicted in Figure 5.

As with the glutamine cycle the glutamine-GABA cycle is not a closed loop. Carbon is lost at the glutamate decarboxylase step. Anaplerosis supplies the missing carbon. Moreover, because glutamate is a key component of both cycles the two cycles are intimately intertwined and sometimes treated as a single entity—the glutamine-glutamate/GABA cycle, e.g., [134,144]. This is because once GABA and glutamate are taken up into astrocytes the carbon skeletons of both compounds enter the TCA cycle and are eventually incorporated into glutamine. Thus, for example, in MR studies of labeling of glutamine in the 4 position in rat brain after administration of cerebral [1-^13^C]glucose it is not possible to determine whether the label in glutamine originated from glutamate or from GABA (ref. [144] and references cited therein).

However, it is possible to distinguish the two cycles by using appropriately-labeled acetate. Classical work by Berl, Van den Berg and colleagues showed that, unlike glucose which is metabolized in both neurons and astrocytes, acetate is metabolized in the small compartment of the brain (*i.e.*, astrocytes) [145,146,147]. (For a review see ref. [148]). MR studies of [^13^C]acetate in brain slices [149] and of neural cells in culture [150,151] have confirmed these earlier studies. Studies of disposition of label derived from [^14^C]acetate in astrocytes and synaptosomes suggest that metabolism of acetate in astrocytes but not in neurons is due to preferential transport [152,153]. Thus, in the brain acetate is metabolized in the same cellular compartment in which carbon atoms originating from neurotransmitter glutamate and GABA are incorporated into glutamine.

This preferential uptake and metabolism of acetate in astrocytes was used by Patel *et al.* [135] as the basis for distinguishing between the glutamine cycle and the glutamine-GABA cycle. Patel *et al.*, infused [2-^13^C]acetate into rats and used MR to detect label in cerebral glutamine and GABA [135]. By comparing the steady-state fractional enrichments of Glu-C4, GABA-C2, and Gln-C4 attained during the infusion of [2-^13^C]acetate to similar measurements following infusion of ]1,6-^13^C_2_]glucose, Patel *et al.*, were able to estimate the relative flux through the glutamine cycle *versus* the glutamine-GABA cycle in rat cerebral cortex [135]. Under 1% halothane anesthesia, cerebral GABA/glutamine cycle flux comprised 23% of total (glutamate plus GABA) neurotransmitter cycling and 18% of total neuronal tricarboxylic acid cycle flux [135].

## 8. Nitrogen Balance in the Glutamine and Glutamine-GABA Cycles

Ingress of either glutamate or GABA into astrocytes results in entry of one nitrogen equivalent, whereas egress of glutamine from astrocytes results in loss of two nitrogen equivalents from these cells. The glutamine cycle in Figure 4 is depicted by red arrows. In the pathway shown carbon mass is balanced but not nitrogen mass. Similarly, in the glutamine-GABA cycle one equivalent of nitrogen enters the astrocytes in the form of GABA, whereas two equivalents exit in the form of glutamine (Figure 5). This raises the question of how nitrogen balance is maintained in the cerebral glutamine- and glutamine-GABA cycles. Various nitrogen shuttles from neurons to astrocytes have recently been analyzed by Calvetti and Somersalo [154]. A commentary of this work has been published [155] and is summarized here. One possible nitrogen shuttle suggested by Calvetti and Somersalo [154] involves the transamination of pyruvate with glutamate catalyzed by alanine aminotransferase in neurons, uptake of alanine by astrocytes, and transamination of alanine in the astrocytes: Equations (30) and (31). However, the specific activity of alanine aminotransferase in the brain is relatively low [156], the rat enzyme has a relatively high *K*_m_ for alanine (~17.5 mM) [157]—much higher than the concentration in normal rat brain of 3.6 nmol/mg protein (≈0.4 mM) [158]—and there is little evidence from ^13^N-labeling studies that this pathway is prominent in normal rat brain [20].

Alanine aminotransferase (neurons): Pyruvate + L-glutamate → L-alanine + α-ketoglutarate (30)

Alanine aminotransferase (astrocytes): L-Alanine + α-ketoglutarate → pyruvate + L-glutamate (31)

Another potential shuttle, first proposed by Hutson and colleagues, involves the branched-chain aminotransferases [159,160]. A branched-chain α-keto acid (e.g., α-ketoisocaproate) is transaminated with glutamate in the neurons to yield leucine and α-ketoglutarate. The leucine is transported into astrocytes where it is transaminated with α-ketoglutarate to yield α-ketoisocaproate and leucine: Equations (32) and (33).

Branched-chain aminotransferase (neurons): L-Glutamate + α-ketoisocaproate → α-ketoglutarate + L-leucine (32)

Branched-chain aminotransferase (astrocytes): L-Leucine + α-ketoglutarate → α-ketoisocaproate + L-glutamate (33)

The possibility that astrocytes metabolize leucine has strong support. For example, as noted above (Section 2.1) branched-chain amino acids are readily taken up across the BBB. Moreover, almost 50 years ago Berl and Frigyesi showed that the leucine in the cat brain is preferentially metabolized in the small compartment (*i.e.*, astrocytes) [161,162]. Other tracer studies with neural cells in culture are consistent with pronounced leucine metabolism in astrocytes [163,164]. Isoleucine is of interest because its transamination in astrocytes will not only replenish glutamate nitrogen in that compartment, but also generate anaplerotic succinyl-CoA [103]. However, flux through this pathway is likely to be modest [103].

An analysis by Rothman *et al.* [165] suggests that a branched-chain amino acid shuttle in brain is feasible. However, as pointed out by these authors [165] the proposed branched-chain amino acid shuttle raises some unresolved issues. The GDH reaction is suggested to proceed in the direction of reductive amination of α-ketoglutarate to glutamate in the neurons, but this is unlikely. Thus, relatively little label derived from intracarotid administration of [^13^N]ammonia is incorporated into rat brain glutamate in MSO-treated rats even when glutamine synthetase is inhibited 85% and the animals are hyperammonemic [14]. Under these conditions, compartmentalization of ammonia metabolism in the brain is disrupted such that blood-derived [^13^N]ammonia, which would normally have been efficiently trapped as glutamine (amide) in astrocytes, freely mixes with the neuronal ammonia pool [14,155]. If the GDH reaction were important for the net synthesis of glutamate in neurons considerable label should have been present in brain glutamate in the MSO-treated rats. The fact that this was not observed suggests that the GDH reaction is not important for the *net* synthesis of glutamate in neurons even under hyperammonemic conditions. Thus, although transfer of leucine and other branched-chain amino acids between neurons and astrocytes is feasible and much evidence suggests that leucine is transaminated in astrocytes, the GDH reaction is unlikely to play a major role in any nitrogen balance mechanism between neurons and astrocytes involving the branched-chain amino acids.

In summary, the major source of the amine moiety of glutamate in astrocytes is still unresolved. As discussed above, a major flow of nitrogen from neurons to astrocytes through alanine is unlikely. One possibility for the origin of glutamate amine nitrogen in the astrocyte is via transamination of α-ketoglutarate with leucine entering across the BBB. As noted above, there is considerable evidence that the leucine carbon skeleton is metabolized in the small compartment; the first step in leucine metabolism is an obligate transamination step. However, the extent to which transamination of blood-derived leucine contributes *directly* to the amine nitrogen in astrocytic glutamate remains unknown, but is likely to be substantial (see below). Another possibility for replenishment of astrocytic glutamate (amine) has been suggested by Pardo *et al.*, based on MR studies of aralar-deficient mice and cultures of neurons and astrocytes derived from these mice [166]. (Aralar is a glutamate-aspartate mitochondrial transporter found predominantly in neurons in the brain) (ref. [166] and references cited therein). Pardo *et al.*, suggest that neuronal aspartate is transported to astrocytes wherein it donates its nitrogen to glutamate via the AspAT reaction [166]. Hertz later suggested a modification [167]. In the Hertz model AspAT-catalyzed transamination of aspartate with α-ketoglutarate in astrocyte cytosol as envisaged by Pardo *et al.* [166] is retained. However, the astrocytic aspartate is generated in the astrocytic mitochondria by transamination of glutamate with oxaloacetate rather than from the mitochondrial AspAT reaction in neurons as envisaged by Pardo *et al.* [166]. The overall Hertz pathway for carbon flow from neuronal-derived aspartate to astrocytic aspartate is summarized in Equation (34) (N, neurons; A, astrocytes; OAA, oxaloacetate). The aspartate generated in the last step of this sequence is obtained by transamination of oxaloacetate with glutamate originating from neurons.

Possible route for transfer of nitrogen from cytosolic neuronal aspartate to astrocytic cytosolic aspartate: Asp (N, cyt) → OAA (A, cyt) → malate (A, cyt) → malate (A, mit) → OAA (A, mit) → Asp (A, mit) → Asp(A, cyt) (34)

We believe that the utilization of AspAT isozymes to provide the amine group in astrocytic glutamate is a very reasonable hypothesis based on the high activity of AspAT in neural cells in both the cytosol and mitochondria. However, despite the elegant suggestions of Pardo *et al.*, and Hertz the question still remains as to what is the *overall* origin of the aspartate/glutamate nitrogen in the brain? As we have discussed above, at least a portion of the astrocyte glutamate nitrogen is obtained from transamination of blood-derived branched-chain amino acids with α-ketoglutarate. Nevertheless, the fact that neurons contain considerable branched-chain aminotransferase activity [23,24] suggests that a portion of the branched-chain amino acids enters the neuronal compartment. In that case, we suggest a modification of the original branched-chain amino acid shuttle as envisaged by Hutson and colleagues [159,160]. The flow of nitrogen according to the branched-chain amino acid shuttle hypothesis of these authors is depicted in Equation (35). However, as we have discussed above, a *net* flow of nitrogen from ammonia to glutamate in neurons is unlikely in normal brain. We propose here a modification of the branched-chain shuttle which incorporates elements of the Pardo *et al.*, and Hertz hypotheses. Thus, we suggest that the presence of branched-chain aminotransferase in astrocytes allows for the direct transfer of the amino group of blood-derived leucine to glutamate in this compartment. On the other hand, the presence of this enzyme in neurons allows for the replenishment of the amine group of glutamate in astrocytes through the sequence shown in Equation (36). Once again, we cannot emphasize enough the metabolic importance of α-ketoglutarate/glutamate-linked aminotransferases in maintaining nitrogen homeostasis in the brain.

Proposed involvement of branched-chain aminotransferases in maintaining nitrogen balance between astrocytes and neurons: Ammonia (N) → Glu (N) → Leu (N) → Leu (A) → Glu (A) (35)

Blood-derived leucine as a possible source of astrocytic glutamate and aspartate nitrogen: Leu (blood) → Leu (N) → Glu (N) → Asp (N) → Asp (A) → Glu (A) (36)

## 9. Disruption of Glutamate Homeostasis in Neurological Diseases

The literature on the role of excess glutamate in neurological diseases is vast. Of necessity, therefore, only a few selected references are presented in this section. As noted in the Introduction glutamate, and to a lesser extent aspartate, are the major excitatory neurotransmitters in the brain. Neurotransmitter glutamate acts upon ionotropic (N-methyl-D-aspartate (NMDA) and α-amino-3-hyroxy-5-methylisoxazole propionic acid (AMPA)) or metabotropic (mGlu1-mGlu8) receptors [168,169]. Excess production of glutamate at the synapse, or inhibition of its reuptake from the synaptic cleft, results in toxicity to adjacent neurons as a result of excessive stimulation of glutamate receptors and calcium overload. Although others had previously noted the neurotoxicity of excess glutamate Olney was the first (almost 50 years ago) to widely publicize the excitoxicity of glutamate [170,171]. In sum, it is critically important to maintain the concentration of extracellular glutamate at a low level to ensure proper neuronal functioning and to prevent excitotoxicity (for some earlier reviews see, for example, ref. [172,173,174,175,176]). Considerable evidence suggests that glutamate excitoxicity plays a prominent pathophysiological role in severe, *acute* insults to the brain [177], including traumatic brain injury [178,179], stroke/ischemia [180,181], epilepsy [182], and perinatal brain injury [183]. However, does excitoxicity contribute to neurodegenerative diseases where neural death occurs slowly? In other words, does *chronic* glutamate excitoxicity also exist? The answer according to Lewerenz and Maher from studies, for example, of appropriate animal models and from downregulation of glutamate transporters is “yes” [177]. Glutamate excitoxicity is suggested to be a prominent factor in slowly progressing neurodegenerative diseases such as AD [184,185], amyotrophic lateral sclerosis (ALS) [186,187,188,189], Huntington disease [190], and Parkinson disease [191,192]. Neuroblastomas represent an intriguing situation in which decreased glutamate transmitter uptake is a contributing factor to neurological disease [80]. This leads to increased extracellular glutamate. Indeed, neoplastic transformation of human astrocytes to malignant gliomas is often associated with seizures and neuronal destruction (ref. [80] and references cited therein).

Based on the importance of glutamate excitotoxicity as a contributing factor to many neurodegenerative diseases, it is not surprising that a great deal of effort has been devoted to the design of small-molecular-weight compounds that can potentially block or lessen the excitoxicity of extracellular glutamate. For example, blockers of ion channels associated with glutamate receptors have been effective in animal models of stroke [193]. Pharmacological activators of EAAT-2/GLT-1 have been explored for decades and are currently emerging as promising tools for protection in a wide variety of neurodegenerative diseases [194,195,196]. In a recent review, Fontana notes that translational activators of EAAT-2/GLT-1, such as ceftriaxone and LDN/OSU-0212320, have significant protective effects in animal models of ALS and epilepsy [194].

Despite these promising leads the literature is littered with descriptions of innumerable treatments designed to block glutamate excitoxicity that were successful in animal models of neurodegeneration, only to fail in clinical trials (reviewed in ref. [197]). Because of this calamitous situation, some researchers have begun to consider an alternative approach to minimizing glutamate excitoxicity other than using traditional pharmacological interventions directed toward glutamate transporters/ion channels—that is, to use an enzyme-based approach. For example, administration of recombinant human AspAT in a rat model of ischemic stroke (middle cerebral artery occlusion) was shown by Pérez-Mato *et al.*, to lower brain and serum glutamate concentrations [198]. The treatment resulted in a reduction in stroke-induced infarct volume and sensorimotor deficit that was most pronounced when oxaloacetate was co-administered with the enzyme [198]. Khanna have championed the idea that interventions designed to upregulate the expression of AspAT in the CNS may be useful in the treatment of diseases associated with glutamate excitotoxicity [197]. Finally, Brusilow and colleagues showed that administration of the glutamine synthetase inhibitor MSO in a mouse model of ALS resulted in a significant increase in survival time [199]. The effect was more pronounced in female mice than in male mice [200]. The authors used an MR technique to show that glutamine and glutamate concentrations in the motor cortex and anterior striatum of the ALS mice were reduced by 60% and 30%, respectively, by the MSO treatment [199]. These findings are consistent with our findings for the effect of MSO on the concentrations of glutamate and glutamine in whole rat brain (Table 2).

## 10. Conclusions

This review highlights the central importance of glutamate as a nitrogen buffer in the brain even in the face of severe hyperammonemia. Nitrogen homeostasis in the brain is maintained in large part by the action of linked glutamate/α-ketoglutarate-dependent aminotransferases. In association with GDH these enzymes provide an efficient means of channeling excess nitrogen from several abundant cerebral amino acids (especially the branched-chain amino acids) toward ammonia. This ammonia and exogenously-derived ammonia are very efficiently incorporated into glutamine as a means of disposing “waste” nitrogen from the brain and as part of the glutamine- and glutamine-GABA cycles. As a result of high activity of glutamine synthetase in astrocytes, but not in most neurons, ammonia metabolism is compartmentalized in the brain. Owing to glutamate-glutamine and GABA-glutamine cycling between neurons and astrocytes there is an input of approximately one nitrogen equivalent into the astrocytes (as glutamate or GABA) from neurons and transfer of approximately two nitrogen equivalents (as glutamine) from astrocytes to neurons. We suggest that glutamate/α-ketoglutarate-linked aminotransferases maintain the amine group of astrocytic glutamate. Especially important enzymes in this regard are the mitochondrial and cytosolic isozymes of AspAT and the branched-chain aminotransferases. Finally, although we have emphasized the fact that glutamate may act as a nitrogen buffer in the CNS excessive glutamate concentrations in the “wrong” compartment may be deleterious. Indeed, glutamate excitoxicity is a contributing factor to many acute and chronic neurodegenerative diseases. Interventions designed to alter key enzyme levels *in vivo* (e.g., MSO administration to inhibit glutamine synthetase; administration of AspAT) and thereby diminish CNS glutamate levels have recently shown promise in animal models of ALS and stroke, respectively. Although in its infancy, we believe that such enzyme-based therapies are an interesting and potentially important new approach to combating glutamate excitoxicity.

## Figures and Tables

**Table 1:** Approximate Concentration (µmol/g Wet weight) of Glutathione and the Most Abundant Amino Acids in the Brain.

	Cat	Rat	Human
Glutamate	7.90 (9.88)	11.6 (14.5)	6.00 (7.50)
Taurine	2.30 (2.88)	6.60 (8.25)	0.93 (1.16)
Glutamine	2.80 (3.50)	4.50 (5.63)	5.80 (7.25)
Aspartate	1.70 (2.13)	2.60 (3.25)	0.96 (1.20)
γ-Aminobutyrate	1.40 (1.75)	2.30 (2.88)	0.42 (0.53)
Glycine	0.78 (0.98)	0.68 (0.85)	0.40 (0.50)
Alanine	0.48 (0.60)	0.65 (0.81)	0.25 (0.31)
Serine	0.48 (0.60)	0.98 (1.23)	0.44 (0.55)
Glutathione	0.49 (0.61)	2.60 (3.25)	0.20 (0.25)

Adapted from [1]. Values in parenthesis are concentrations (mM) assuming a water content of 80%.

**Table 2:** Metabolite Concentrations (mmol/kg wet eight) in Rat Cerebral Cortex During Ammonia-Induced and L-Methionine-*S*,*R*-Sulfoximine (MSO)-Induced Intoxication.

Metabolite	Control (NaAc-Infused)	NH_4_Ac-Infused	MSO-Treated + NaAc-Infused	MSO-Treated + NH_4_Ac-Infused
Ammonia (blood)	0.191 ± 0.063	0.710 ± 0.150 *	0.432 ± 0.089 *	1.02 ± 0.07 *
Ammonia	0.326 ± 0.063	0.985 ± 0.084	0.855 ± 0.031	2.48 ± 0.06
α-Ketoglutarate	0.096 ± 0.011	0.112 ± 0.011	0.157 ± 0.006 *	0.141 ± 0.018 *
L-Glutamate	12.9 ± 0.4	11.1 ± 0.3 *	10.1 ± 0.3 *	11.6 ± 0.4 *^,†^
L-Glutamine	8.04 ± 0.019	18.5 ± 0.8 *	3.79 ± 0.54 *	6.97 ± 1.00 *

Adult male Wistar rats were anesthetized and catheters were inserted into a tail artery and one tail vein. The rats were tracheotomized, curarized and passively ventilated with 30% O_2_–70% N_2_O. Blood temperature and acid-balance were maintained within normal limits. Animals that received MSO were injected with 150 mg/Kg (a dose designed to substantially inhibit glutamine synthetase by >90% but not to induce seizures) 2.0–2.5 h before infusions were begun. The rats were then infused intravenously with either 3 M sodium acetate (NaAc) or 3 M ammonium acetate (NH_4_Ac) for 2 h at a rate of 6.2 μL/min, after which times the animals were euthanized by freezing the brains *in situ* with liquid N_2_. The brains were removed, powdered under liquid N_2_, weighed at −20 °C and extracted with 3 M perchloric acid. Metabolites in the neutralized perchlorate extract were analyzed as described in ref. [95]. *n* = 4 or 5 in each group. * *p* < 0.01 by Dunett’s test for multiple comparisons; ^†^ different from the value obtained with the MSO/NaAc-treated rats with *p* < 0.05 by the Student t test. Modified from ref. [102].

## Abbreviations

The following abbreviations are used in this manuscript:

AD: Alzheimer disease
ALS: Amyotrophic lateral sclerosis
AspAT: Aspartate aminotransferase
BBB: Blood brain barrier
BCAT: Branched-chain aminotransferase
BCATc: Cytosolic isozyme of BCAT
BCATm: Mitochondrial isozyme of BCAT
CNS: Central nervous system
CSF: Cerebrospinal fluid
cyt: Cytosolic
GABA: γ-Aminobutyrate
GDH: Glutamate dehydrogenase
GLAST: Glutamate aspartate transporter
GSH: Glutathione
MR: Magnetic resonance (also nuclear magnetic resonance)
Mit: Mitochondrial
MSO: L-Methionine-*S*,*R*-sulfoximine
5-OP: 5-Oxoproline
PNC: Purine nucleotide cycle
PrP: wild type prion protein
TCA: Tricarboxylic acid
